# Exploiting the Therapeutic Potential of Endogenous Immunomodulatory Systems in Multiple Sclerosis—Special Focus on the Peroxisome Proliferator-Activated Receptors (PPARs) and the Kynurenines

**DOI:** 10.3390/ijms20020426

**Published:** 2019-01-19

**Authors:** Bernadett Fakan, Levente Szalardy, Laszlo Vecsei

**Affiliations:** 1 Department of Neurology, Faculty of Medicine, Albert Szent-Györgyi Clinical Center, University of Szeged, H-6725 Szeged, Semmelweis u. 6, Hungary; bernadett.fakan@gmail.com (B.F.); szalardy.levente@med.u-szeged.hu (L.S.); 2 MTA-SZTE Neuroscience Research Group, H-6725 Szeged, Semmelweis u. 6, Hungary

**Keywords:** kynurenines, multiple sclerosis, peroxisome proliferator-activated receptor, PGC-1α, PPAR

## Abstract

Multiple sclerosis (MS) is a progressive neurodegenerative disease, characterized by autoimmune central nervous system (CNS) demyelination attributable to a disturbed balance between encephalitic T helper 1 (Th1) and T helper 17 (Th17) and immunomodulatory regulatory T cell (Treg) and T helper 2 (Th2) cells, and an alternatively activated macrophage (M2) excess. Endogenous molecular systems regulating these inflammatory processes have recently been investigated to identify molecules that can potentially influence the course of the disease. These include the peroxisome proliferator-activated receptors (PPARs), PPARγ coactivator-1alpha (PGC-1α), and kynurenine pathway metabolites. Although all PPARs ameliorate experimental autoimmune encephalomyelitis (EAE), recent evidence suggests that PPARα, PPARβ/δ agonists have less pronounced immunomodulatory effects and, along with PGC-1α, are not biomarkers of neuroinflammation in contrast to PPARγ. Small clinical trials with PPARγ agonists have been published with positive results. Proposed as immunomodulatory and neuroprotective, the therapeutic use of PGC-1α activation needs to be assessed in EAE/MS. The activation of indolamine 2,3-dioxygenase (IDO), the rate-limiting step of the kynurenine pathway of tryptophan (Trp) metabolism, plays crucial immunomodulatory roles. Indeed, Trp metabolites have therapeutic relevance in EAE and drugs with structural analogy to kynurenines, such as teriflunomide, are already approved for MS. Further studies are required to gain deeper knowledge of such endogenous immunomodulatory pathways with potential therapeutic implications in MS.

## 1. Introduction

Multiple sclerosis (MS) is a chronic progressive neuroinflammatory and neurodegenerative disease, characterized by demyelination, and axonal and neuronal degeneration in the central nervous system (CNS), mediated in part by target-specific autoimmune processes [1]. As the etiology and exact pathogenesis of MS is still incompletely understood, experimental models have been developed to gain a better understanding of the disease. The most widely used model is experimental autoimmune encephalomyelitis (EAE), which is an inflammatory and demyelinating autoimmune condition of rodents (predominantly mice), caused by an immune response against injected CNS myelin constituents, such as myelin basic protein (MBP), proteolipid protein, or myelin oligodendrocyte glycoprotein (MOG), or a passive transfer of cluster of differentiation 4-positive (CD4+) or cluster of differentiation 8-positive (CD8+) encephalitogenic T cells [1,2]. Based predominantly on data gained from animal models [3], it is now supposed that T helper 1 (Th1) and T helper 17 (Th17) lymphocytes (expressing interleukin (IL)-1 and IL-17, respectively) play a major role in the development of the condition [4], with concomitant decreases in T helper 2 (Th2) [5] and regulatory T cell (Treg) responses having been heavily implicated as well [6]. The anti-inflammatory IL-4 and IL-10 as well as Th2 cells, which control the development and function of Th1 cells, are considered to be important in the recovery phase of the disease [5], whereas Tregs play an inhibitory role in the late stages of EAE immunopathogenesis [6]. There is also an increasing evidence on the role of the pro-inflammatory classically-activated macrophage (M1) bias of the profile of infiltrating macrophages [7] with increased IL-6 as well as IL-12 cytokine levels having been implicated [4,8], whereas M2 polarized microglia/macrophages are supposed to play key roles in remyelination [9]. IL-12 is a macrophage/microglia derived pro-inflammatory cytokine that has a role in the pathogenesis of CNS demyelination in EAE/MS [10] also by participating in the differentiation of Th1 cells [11]. Though IL-6 has both pro- and anti-inflammatory properties, IL-6-deficiency has been repeatedly reported to render mice resistant to EAE with consistent shifts towards Th2 responses [12,13,14]. Furthermore, IL-6 appears to play central roles in the development of Th17 cells and the downregulation of Tregs [4]. Demyelination, axonal degeneration, and the consequential permanent disability in MS patients are results of the excessive production of these inflammatory cytokines and reactive oxygen species (ROS) produced by infiltrating macrophages and resident microglia [15,16,17,18]. In addition to the direct cytotoxicity exerted on myelin-producing oligodendrocytes and demyelinated axons, this process leads to astrocyte activation, which plays a dual role in MS, as reactive astrocytes both stimulate and restrain inflammation and tissue damage [19], in part by increasing the leakage of the blood-brain barrier (BBB) and by secreting immunosuppressive molecules, respectively [20]. In addition, demyelination is also supposed to be attributable, at least in part, to excitotoxicity, based on the observations that the blockade of α-amino-3-hydroxy-5-methyl-4-isoxazolepropionic acid (AMPA)/kainate receptors present on oligodendrocytes diminish symptoms of EAE [21]. 

A number of different endogenous molecular systems have been implicated in the regulation of such autoaggressive processes by promoting a shift from the encephalitogenic/demyelinating phenotype of the immune response (i.e., Th1, Th17, and M1 cells) to enable repressive and restorative responses (i.e., mediated by Th2, Treg, and M2), thus serving as innate suppressors of target-specific immunity. The recognition of such processes is significant, first as the increased expression/production of their contributors may serve as biomarkers with clinical relevance in different body compartments (i.e., prognostic biomarkers or indicators of a therapeutic effect in the blood or cerebrospinal fluid (CSF)), and their manipulation might provide novel therapeutic targets and soil for future research and drug development.

This review summarizes the current state of knowledge about certain endogenous molecular pathways with the potential ability to influence the pathogenesis and/or course of MS, with special emphasis on peroxisome proliferator-activated receptors (PPARs), PPARγ coactivator-1alpha (PGC-1α), and the kynurenines. Highlights are given on conceptual-, experimental-, and clinical-phase therapeutic approaches based on the alterations of the discussed mechanisms. The literature search was conducted within the PubMed database by using the keywords ‘peroxisome proliferator-activated receptor’ or ‘PPAR’ or ‘PGC-1’ or ‘kynurenine’ or ‘tryptophan’ or ‘IDO’ and ‘multiple sclerosis’ or ‘MS’ or ‘experimental autoimmune encephalomyelitis’ or ‘experimental allergic encephalomyelitis’ or ‘EAE’. Reviews and original research reports were both considered along with relevant references originally undetected by the search engine, but cited within the identified publications.

## 2. The PPAR System and PGC-1α

PPARs are members of a nuclear hormone receptor superfamily of ligand-activated transcriptional factors [22], with well-studied regulatory roles in lipid and glucose homeostasis and adipocyte differentiation [23]. PPARs alter target gene expression by binding to peroxisome proliferator response elements (PPREs) after becoming activated by fatty acid intermediates [24]. Encoded by different genes [25], PPARs have three subtypes, including PPARα, PPARβ/δ, and PPARγ, which have different, though, in part, overlapping, effects on cellular physiology and show different specificity in terms of their ligand-binding properties (Table 1) [26].

### 2.1. In Vitro Basis of the Role of PPARs and PGC-1α in Neuromodulation

Out of the three subtypes of PPARs, PPARγ is the most extensively examined. PPARγ was originally studied for its role in lipid and glucose metabolism, with its preferential ligands, the thiazolidinediones, currently being widely used in the pharmacological management of type 2 diabetes mellitus (e.g., pioglitazone, Table 1). In addition to adipocytes and hepatocytes, PPARγ has more recently been shown to be expressed in neurons [45], macrophages [46], oligodendrocytes [47], astrocytes [48], T [49] and B [50] lymphocytes, dendritic cells (DCs) [51], and brain endothelial cells as well [52]. Furthermore, PPARγ has recently been identified as an important factor in the regulation of the organ-specific immune response [53]. Indeed, its activation has been shown to suppress the expression of inflammatory cytokines in astrocytes and macrophages/microglia [22,35,54], to inhibit the expansion of Th1 [10,55], Th17 [55,56], and B cells [50], and to the promote a switch to Treg [55] and Th2 [57,58] responses. Of note, PPARγ is markedly upregulated in activated T cells [46,59,60] and macrophages [22], and ligand activation of PPARγ in these cells has been associated with the suppression of their responses, including the apoptosis of activated cells [49,61]. In addition, adoptive transfer of PPARγ-activated antigen-presenting DCs were reported to result in CD4+ T lymphocyte anergy [62]. Furthermore, ligand activation of PPARγ has been reported to also decrease the transmigration of encephalitogenic T lymphocytes through activated brain endothelial cells, expanding its potential implications in protecting against T cell-mediated autoimmune CNS processes [52]. More recently, studies on human material demonstrated an exclusive role of PPARγ among all PPARs in promoting the differentiation of macrophages into an M2 profile [63]. Indeed, the expression of PPARγ selectively correlated with M2 phenotypic markers in human atherosclerotic lesions [63], and the upregulation of PPARγ (but no other PPARs) has been documented during the M2 switch due to IL-4 induction in human macrophages [63,64]. This is in accordance with serial reports demonstrating increased PPARγ expression upon IL-4 stimulus in microglia [65] and macrophages [22,66,67,68]. Furthermore, PPARγ has been demonstrated to be upregulated in an antigen-induced in vitro model of demyelination [69], likewise suggesting an endogenous protective role of this isoform in demyelinating diseases, such as MS. In addition, ligand activation of PPARγ stimulated oligodendrocyte differentiation from neural stem cells [70], promoted and accelerated the differentiation of oligodendrocyte progenitor cells in vitro with an additional increase in antioxidant defenses [71,72], and increased lipid production and terminal differentiation of cultured oligodendrocytes [73], together suggesting an additional possible protective role of PPARγ in MS as a mediator of remyelination. Recent evidence suggests that the promotion of remyelination by PPARγ may be mediated at least in part by its regulatory effects on NF-κB and Wnt/β-catenin pathways [74]. The neuroprotective effects of PPARγ have also been widely documented in vitro in various experimental paradigms of neurodegeneration, broadening its potential therapeutic perspectives in MS [45,75,76,77,78,79].

PPARα is important in the regulation of fatty acid metabolism [80] and responses to ROS [81], and its primary exogenous ligands, the fibrates (e.g., fenofibrate, with preference to the PPARα isoform, Table 1) are widely used in medicine to decrease circulating triglyceride levels. Unlike other isotypes, PPARα is not expressed in oligodendroglia [47,82,83,84], but is present in astrocytes (where it is the predominant isoform) [84,85], neurons [84,86], myeloid cells [46,61,87], and T and B lymphocytes [46]. Notably, however, contrasting with PPARγ, PPARα is markedly downregulated in T cells upon activation [46,59]. PPARα is also known for inducing apoptosis in macrophages [8,61]. However, the ability of PPARα to induce apoptosis in activated T cells has been questioned [49], and some of its demonstrated anti-inflammatory effects have been linked preferentially to males [88], contrasting with the more recently reported female predominance of PPARγ to produce IL-17A in T cells [89]. Likewise, the expression of PPARα during IL-4-stimulated M2 activation was found only mildly increased (to a remarkably smaller extent compared to PPARγ) [68] or largely unchanged [63,64,90] in studies performed on human monocytes; no data is at present available in this respect using cell lines from other species. These together suggest somewhat less pronounced possibilities for PPARα as an endogenous immunomodulator compared to PPARγ, which tends to be in line with multiple observations that certain anti-inflammatory actions of fibrates appear to be in fact PPARα-independent [32,47,59]. Likewise, though being well-known as a potent PPARα activator, gemfibrozil has recently been shown to induce the expression of myelin-specific genes in both PPARα +/+ and PPARα −/− mice, but in a PPARβ/δ-dependent manner, and increased the expression of PPARβ/δ (but not PPARα or PPARγ) in oligodendrocytes and mixed glial cultures [47].

The expression of PPARβ/δ, the most abundant PPAR isotype in the brain [84,85], is linked predominantly to oligodendrocytes [47,82,91] and neurons [84,85,86,91]. Though the exact functions of PPARβ/δ have not yet been clarified in detail, the available evidence indicates that it promotes myelin production in oligodendrocytes [26], and its ligand activation increases the number of myelin-producing oligodendrocytes [92]. To date, we are not aware of reports on altered expression of PPARβ/δ in activated T lymphocytes. However, PPARβ/δ-deficient T cells (but not B cells) were reported to be hyperresponsive to stimulation, including an increased expression of IL-17A in PPARβ/δ knockout T lymphocytes [24], whereas PPARβ/δ ligation led to decreased IL-17 expression among similar Th17-polarizing conditions and decreased interferon-γ (IFNγ) production among Th1-polarizing conditions [11] (others found indifferent IFNγ production using another ligand [43]). Though PPARβ/δ seems to be indispensable for M2 activation in rodents [93,94], the relevance of this feature in humans has been questioned by recent studies on human cell lines suggesting an exclusive role of PPARγ in this respect, with the expression of PPARβ/δ having been found either unaltered [64,90] or decreased in human M2 cells [63]. Notably, a specific pharmacological ligation of PPARβ/δ, though it demonstrated some anti-inflammatory action, failed to protect against antigen-induced demyelination in vitro [95] and likewise failed to inhibit the antigen-induced proliferation of CD4+ T lymphocytes in vitro and ex vivo [11]. 

PGC-1α is a pan-coactivator of the PPARs, the expression of which is enhanced in turn by a number of different PPAR ligands [96,97,98]. In addition to being a master regulator of cellular lipid and glucose metabolism, PGC-1α is essential in maintaining energetic homeostasis and cell viability by limiting ROS-induced damage and enhancing adaptive mitochondrial biogenesis and oxidative phosphorylation [99,100,101]. In line with these, PGC-1α deficiency has been linked to the pathogenesis of various neurodegenerative disorders in humans where mitochondrial dysfunction and excessive ROS production has been implicated in the pathogenesis, and its murine knockouts have been proposed as viable models of mitochondrial encephalopathy [102,103,104]. The in vitro evidence that links PGC-1α activation to immunomodulation includes its capability to diminish the expression of IL-6 [105], which is in line with the remarkable increase of IL-6 observed in PGC-1α knockout mice and the inverse association between PGC-1α and IL-6 levels in human diabetic muscles [106]. In addition, PGC-1α has been shown to potently decrease the expression of IL-12 in the skeletal muscle of mice, skewing the M1/M2 balance to an anti-inflammatory M2 phenotype [107]. PGC-1α expression has also been demonstrated in macrophages [108], granulocytes, and lymphocytes [109], and its expression was found to be enhanced after B cell receptor activation in lymphocytes [110].

### 2.2. In Vivo and Human Implications for a Protective Role of PPARs and PGC-1α in EAE/MS

**Figure 1:**
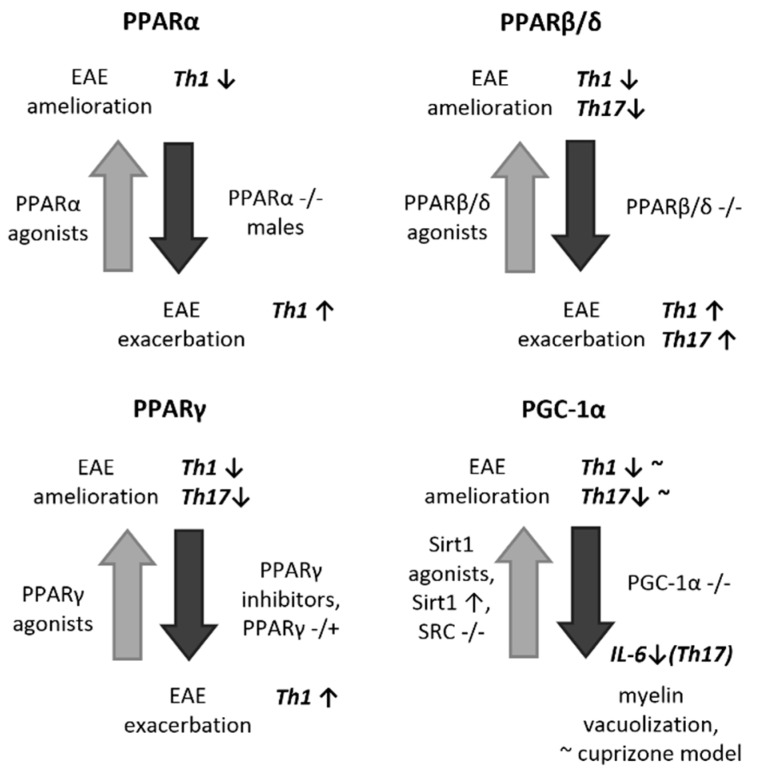
Schematic representation of the available literature data indicating the role of PPARs and PGC-1α in EAE. EAE: experimental autoimmune encephalomyelitis, IL-6: interleukin-6, PPAR: peroxisome proliferator-activated receptor, Sirt1: Sirtuin (silent mating type information regulation 2 homolog) 1, SRC: steroid receptor coactivator, Th: T helper lymphocyte. Large bold arrows point towards the phenotype resulted by the pharmacological or genetic interventions (gray up arrows point to amelioration of EAE, black down arrows point to exacerbation of EAE or relevant similar pathology). Small up and down arrows represent increase and decrease in number, respectively. Tilde (~) used after a term represent ‘insufficient or contrasting evidence’, whereas it represents ‘resemblance’ when used before a term.

In line with the mechanisms described above, experimental observations indicate that ligand activation of PPARγ renders protection against EAE in mice [26,35,39,69,70,111,112], with the proposed/demonstrated mechanisms involved including the ability to alter astroglial and macrophage/microglial cytokine production as well as the Th1-Th17/Th2-Treg balance (Figure 1). Similarly, moringin, a.k.a. glucomoringin isothiocyanate, has also been found to be protective in EAE, acting as a modulator of neuroinflammation by upregulating the Wnt/β-catenin pathway and leading to subsequent overexpression of PPARγ [113,114], suggesting that the protective immunomodulatory effects of Wnt/β-catenin pathway activators in EAE [115] may be at least in part mediated by PPARγ. Importantly, in addition to a preventive effect in the case of a pretreatment or a simultaneous treatment with PPARγ agonists, ligand activation of PPARγ with pioglitazone was also proven effective when provided after EAE disease onset, underlining its therapeutic potential [26]. In line with all these, the reports are largely concordant regarding the effect of genetic deficiencies in [116,117,118] or the pharmacological inhibition of [117,118] PPARγ to aggravate the course of EAE in rodents. In addition, an elevated expression of PPARγ has been demonstrated within the CNS lesions of EAE mice [25,32,114,119], suggestive of an endogenous anti-inflammatory response to counteract autoimmunity. At the human level, a similar increase in PPARγ levels has recently been described in the CSF of patients with definite MS, correlating with inflammatory CSF alterations, such as IgG index and leukocyte cell count, and demonstrating an association with increased disease severity [120].

Experimental results with other PPARs are less concordant and conclusive (Figure 1). Notably, homozygous deficiency in PPARα in mice likewise resulted in exacerbated hallmark features of EAE at multiple levels of investigation; however, only in male subjects, being concordant with in vitro results [88]. Although pharmacological application of potent PPARα agonists resulted in a decreased severity of EAE in mice [32,33,88,121], even when administered after EAE onset [33,121], it is uncertain to what extent this effect was indeed mediated by PPARα-dependent processes [32,47]. Of note, however, the expression of PPARα at the mRNA level was shown to be downregulated in the cerebellum of mice with EAE, contrasting with the increased expression of other PPAR isoforms [32], suggesting that its pathway may not be activated upon CNS inflammatory stimuli as an endogenous regulatory response. In line with this, no increase was detected in the CSF levels of PPARα in MS patients [122].

Despite the paucity and heterogeneity of supportive in vitro data, pharmacological ligands of PPARβ/δ has repeatedly led to clinical amelioration of EAE in mice [11,43,92]. Contrasting with PPARγ, this effect is especially pronounced when administered at later stages of the disease course (i.e., during already established EAE), suggesting prominent roles in recovery, in particular in remyelination, in addition to the immunomodulatory effects [43,92]. Likewise, PPARβ/δ-deficient mice developed a more severe or prolonged EAE [24,123], and the supportive findings implicated PPARβ/δ in the endogenous modulation of astrocytic and microglial inflammatory reactions [43,124], the promotion of Th2 and Treg responses, and the regulation of Th1 and Th17 responses [11] (Figure 1). As regards PPARβ/δ expression in EAE, the mRNA levels of PPARβ/δ were reported to be increased in the cerebellum of EAE mice [32]; however, no alteration in PPARβ/δ expression could be observed by others in the spinal cord (with only a modest increase in the spleen) [124]. Of human relevance, increased expression of a set of PPARβ/δ-dependent genes has been reported within demyelinating lesions in MS brains, with particular regards to myelin-degrading macrophages [125]. Data on the expression PPARβ/δ itself in the human MS brain has not yet been reported; however, no alteration in the CSF levels of PPARβ/δ at the protein level could be detected in MS compared to non-inflammatory controls [122].

The administration of ultrapurified anti-inflammatory eicosapentaenoic acid (EPA), one of the n-3 polyunsaturated fatty acids (PUFAs), has been found to alleviate the clinical course of EAE while increasing the production of all PPAR isotypes in CNS-infiltrating CD4+ T cells and diminishing IL-17 production [126]. Its effect is partly explained by the ability of EPA to cross the BBB via simple diffusion, and thereby to inhibit re-stimulation of autoreactive CD4+ T cells in the perivascular space [127,128], and partly by increasing Treg cell and reducing Th17 cell activity through the activation of PPARγ [129].

In support of a protective potential of PGC-1α in EAE/MS, treatment of mice with resveratrol, a widely used inducer of its expression through the activation of Sirtuin 1 (SIRT1) [99], has been linked to clinical improvements in relapsing-remitting acute [130] as well as in chronic EAEs [131,132,133]. However, contrasting results have also been published demonstrating worsening of both EAE and virus-induced spinal demyelination by resveratrol [134]. Other SIRT1 activating compounds or formulations have demonstrated beneficial effect on the spinal cord demyelinating lesions in EAE and in a virus-induced in vivo model of MS [135] and/or conferred protection against retinal ganglion cell (RGC) loss in EAE and virus-induced optic neuritis in rodents [130,131,135,136]. Notably, the findings suggested that these results in the spinal cord and retina were preferentially attributed to neuroprotection rather than anti-inflammatory features. However, transgenic overexpression of SIRT1 likewise ameliorated the behavioral signs of chronic EAE, accompanied by neuronal protection and diminished demyelination in the spinal cord, along with an enhancement of Th2 and suppression of Th1 and Th17 responses in the white matter of the spinal cord in this report [137] (Figure 1). Furthermore, SIRT1 has been shown to be upregulated in the spinal cord neurons of EAE mice, suggesting an endogenous neuroprotective response as well [137,138]. The literature is, however, rather contradictory about SIRT1 per se as well, since a study reported the amelioration of the features of EAE either by knockout out or pharmacologically blocking SIRT1 [139]. Less directly, but rather in support of a protective potential of PGC-1α, a dampened disease course of EAE was reported in steroid receptor coactivator-3 (SRC-3)-deficient mice (a deficiency previously reported to promote the activation of PGC-1α); however, these effects were proposed to be attributed to a PPARβ/δ-mediated M2 activation of microglia by the authors [124]. To date, no studies have been reported on the influence of a direct PGC-1α deficiency or overexpression on EAE in mice. Although no definite focal demyelination or inflammation could be detected in the CNS of PGC-1α-deficient animals as seen in EAE, the knockout mice develop dysmyelination [140] and oligodendroglial and intramyelinic vacuolation [102,103] strikingly reminiscent of that seen in mice treated with the toxin cuprizone used in the in vivo modeling of MS [141]. With human relevance, the potent PGC-1α activator, SIRT1, has recently been found to be expressed in both acute and chronic plaques of MS brains, co-localizing with T helper lymphocytes, cells with the monocyte lineage, oligodendrocytes, and astrocytes, and a significant decrease of SIRT1 expression in peripheral blood mononuclear cells (PBMCs) during relapse has been documented [142]. As regards PGC-1α itself, its increased expression was reported in astrocytes within active MS lesions [100,105]. Contrastingly, however, the same group published a consistent decrease in PGC-1α expression in the cortex of patients with MS [143]. No alteration was found in the CSF of patients with MS in PGC-1α expression at the protein level, however, suggesting no use of this molecule as a biomarker [122].

### 2.3. Therapeutic and Diagnostic Perspectives of PPARs and PGC-1α in MS

As a sum of the above, the most concordant findings regarding EAE/MS can be linked to PPARγ, implicating its potential role as an endogenous immunomodulatory molecule as well as a biomarker with potential human clinical relevance. To date, only scattered evidence is available at the clinical level for a potential therapeutic value of PPARγ agonists in MS, with reports available exclusively about pioglitazone, a drug approved by the Food and Drug Administration (FDA) for the treatment of type 2 diabetes mellitus. A single case report about treatment of a secondary progressive MS patient with daily treatment of 45 mg of pioglitazone p.o. reported stable disease with subtle clinical improvement after 3 years [144]. A subsequent small (*n* = 11 vs. 10) randomized controlled trial (RCT) compared the safety and efficacy of pioglitazone (30 mg p.o. daily) with placebo as an add-on to interferon β-1α (IFNβ-1α) in relapsing-remitting MS patients after 1 year. No difference in terms of adverse events or clinical progression could be detected; however, the magnetic resonance imaging (MRI) follow-up demonstrated a significant reduction in gray matter atrophy and a tendency for a decreased lesion load in the group treated with pioglitazone [145]. Another MRI study applying diffusion tensor imaging (DTI) revealed that pioglitazone reduced the conversion of normal appearing white matter into MS lesions during the 1-year follow-up of relapsing-remitting MS patients [146]. Most recently, a prospective cohort study on obese MS patients compared pioglitazone (15–30 mg per day) with placebo (*n* = 10 vs. 20, respectively) after 6 months of follow-up, revealing a significant decrease in the number of new or enlarging T2 lesions and contrast-enhancing lesions compared to controls, in association with better outcomes in terms of serum biomarkers, such as lower leptin and adiponectin levels and a higher Treg count [147]. These results highlight the need of further, larger scale clinical trials to evaluate the efficacy and potential utility of this safe and well-known molecule already approved with other indications. The potential use of CSF or serum PPARγ protein levels as a prognostic or surrogate marker (i.e., of treatment efficacy) in MS merits further supportive studies.

Though EPA, a paninducer of PPARs, showed promise in EAE experiments, Torkildsen et al. reported no beneficial clinical effects in MS on disease progression either in monotherapy or as an add-on to INFβ, based on the results of the ω-3 fatty acid treatment in multiple sclerosis (OFAMS) Study [148].

Despite the indifferent findings in the human CSF and the lack of a potential role as a biomarker, the involvement and potential therapeutic aspects of PGC-1α needs to be directly assessed in EAE/MS, due to its proposed immunomodulatory and neuroprotective features.

## 3. The Kynurenine Pathway

Tryptophan (Trp), an essential amino acid, is the starting molecule for the synthesis of serotonin and l-kynurenine. Although the serotonin pathway and its functions are more emphasized in the academic and educational literature, 95% of Trp is in fact metabolized through the kynurenine pathway (KP), eventually providing NAD+ for further metabolic processes [149,150]. The rate-limiting step of the pathway is the conversion of Trp to *N*-formyl-l-kynurenine by indolamine 2,3-dioxygenase (IDO) in extrahepatic tissues and Trp 2,3-dioxygenase (TDO) in the liver, which is followed by formamidase-mediated catabolism of *N*-formyl-l-kynurenine to yield l-kynurenine, the central molecule of the KP. Depending on whether the kynurenine aminotransferase (KAT), the kynure nine 3-monooxygenase (KMO), or the kynureninase enzymes become more activated or expressed (depending on cell type and pathological conditions), l-kynurenine is metabolized into kynurenic acid (KYNA), 3-hydroxy-l-kynurenine (3-HK), and through anthranilic acid (AA), respectively. Both AA and 3-HK can be metabolized into 3-hydroxyanthranilic acid (3-HAA), that can be further converted by 3-hydroxyanthranilate oxidase (3-HAO) to yield quinolinic acid (QUIN), which is the source of NAD+ production [151]. 

KYNA has long been known as an antagonist of ionotropic glutamate receptors, such as N-methyl-D-aspartate (NMDA), kainate, and AMPA receptors [152,153]. Due to its potential to inhibit glutamate-induced excitotoxicity and to decrease the excitotoxic effect of QUIN [154], and to its more recently identified direct potent antioxidant features [155], it has been implicated as an anticonvulsive and neuroprotective molecule. QUIN is an NMDA receptor agonist and has a key role in energy homeostasis at physiological, low nanomolar concentrations; however, in pathophysiological conditions, QUIN overproduction induces cellular toxicity by multiple mechanisms, predominantly by enhancing glutamate excitotoxicity and ROS production [156,157,158]. Regarding 3-HK, there is increasing evidence demonstrating that it is neurotoxic, promotes cell death, and has a synergistic neurotoxic effect with QUIN [159,160]. However, its function is largely NMDA receptor-independent and is linked preferentially to ROS production partly through its catabolite, 3-HAA [161], which has carcinogenic properties and readily auto-oxidizes while producing ROS [162].

### 3.1. In Vitro Basis of the Role of the Kynurenine Pathway in Neuromodulation

**Figure 2:**
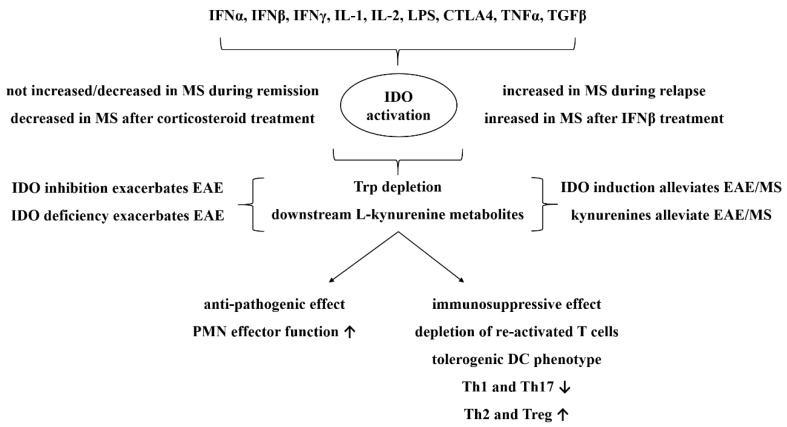
Schematic depiction of potential and demonstrated roles of indolamine 2,3-dioxygenase (IDO) activation and downstream l-kynurenine metabolites inflammation, with particular focus on EAE and MS. CTLA4: cytotoxic T-lymphocyte-associated protein 4, EAE: experimental autoimmune encephalomyelitis, DC: dendritic cell, IDO: indolamine 2,3-dioxygenase, IFN: interferon, IL: interleukin, LPS: lipopolysaccharide, MS, multiple sclerosis, PMN: polymorphonuclear cell, TGFβ: transforming growth factor β, Th: T helper lymphocyte, Treg: regulatory T lymphocyte, TNFα: tumor necrosis factor α, Trp: tryptophan. Up and down arrows represent increase and decrease, respectively.

In addition to the ’canonical’ neurotoxic/neuroprotective features of the kynurenine metabolites, emerging studies from the past decade have provided evidence for KP to have important modulatory roles in innate and adaptive immune responses as well [163]. This is predominantly attributable to the activation of its rate-limiting enzyme, IDO, which (contrasting with the constitutively expressed TDO in the liver) is activated in various cell types upon inflammatory stimuli, such as IFNα, IFNβ, IFNγ, tumor necrosis factor alpha (TNFα), transforming growth factor-β (TGFβ), IL-1, IL-2, cytotoxic T lymphocyte antigen 4 (CTLA4), and lipopolysaccharide (LPS) [151,164]. The effect of IDO during immune responses was first observed by Pfefferkorn et al., who found that IFNγ treatment evoked a significant antitoxoplasma effect in vitro in correlation with Trp depletion in the medium [165]. Since then, research has shown that the immunological effects of IDO are dichotomous. Although IDO activation has potent antimicrobial effects and supports the function of polymorphonuclear cells, it is also associated with potent immunosuppressive effects, participating in the promotion of immunotolerance both in physiology and disease [151] (Figure 2). This effect is realized by inhibiting and depleting both CD8+ and CD4+ Th1 lymphocytes, thus shifting the Th1-Th2 balance towards the Th2 response [166] and promoting Treg generation from T cell precursors, while compromising Th17 formation [167]. These effects are attributed in part by the depletion of Trp and in part by the action of different KP metabolites, especially l-kynurenine, 3-HK, 3-HAA, and probably QUIN, and are thought to be predominantly mediated by plasmocytoid DCs developing a tolerogenic phenotype [151]. Indeed, 3-HK and 3-HAA (and less consistently QUIN) have been linked to the inhibition of T cell proliferation and the apoptotic depletion of activated Th1 cells [168,169,170,171,172,173]. In addition to the combined effect of Trp depletion and the presence of downstream kynurenine metabolites on Treg promotion [174], l-kynurenine has been reported to per se promote Treg development, by an agonistic action on aryl hydrocarbon receptor (AHR), a ligand-activated transcription factor known as the receptor of the famous pollutant, 2,3,7,8-tetrachlorodibenzo-p-dioxin (TCDD). This study identified l-kynurenine as an endogenous agonist for AHR [167]. Ligand activation of AHR has been reported to contribute to Treg development and Th17 suppression both on T cells and pDCs [175,176]; however, it has also been demonstrated to activate IDO in DCs [177], suggesting a forward loop in kynurenine-induced AHR activation. It should also be noted, however, that not all AHR agonists result in Th17 suppression and Treg development, with certain ligands peculiarly causing an opposite outcome [178,179]. IDO-mediated immunosuppression with the contribution of downstream kynurenine metabolites are considered to be essential in the development of materno-fetal tolerance, allograft acceptance, tumor camouflage, and human immune deficiency virus (HIV)- HIV-induced acquired immune deficiency syndrome (AIDS) [151].

The immunomodulatory potential of KYNA, the end-metabolite of the KAT branch of the KP, is less well examined and established, but it may involve the downregulation proinflammatory cytokines, IL-6 [180,181] and TNFα [181]. Of note, KYNA has also been identified as a potent agonist of the AHR [180,182]; however, studies directly demonstrating the hence plausible AHR-mediated Treg/Th17 modulating effect of KYNA are lacking. Despite this, KYNA has very recently been reported to decrease IL-17 expression in activated T cells and to deplete Th17 cells in another way, namely by acting on G-protein-coupled receptor 25 (GPR35) on DCs, suppressing their IL-23 production [183].

In addition to that fact that both the PPAR/PGC-1α and the kynurenine pathway system represent endogenous molecular apparatuses with potent immunoregulatory potency, a direct link has recently been proposed between the PPAR/PGC-1α system and kynurenine metabolism. Indeed, a comprehensive study investigating the influence of PGC-1α on depressive-like behavior in mice reported increased and decreased expression of the KAT enzyme genes in tissues overexpressing or being deficient in PGC-1α, respectively [184]. The authors also found that PPARα and PPARβ/δ ligand activation led to similar increases in KAT activity in myotubes, which was largely dependent on the presence of PGC-1α (albeit not consistently for all KAT isoforms in the case of PPARα activation). On the other hand, silencing of these PPARs led to a decreased expression of KAT enzymes (most consistently for PPARα). As the potential of PGC-1α overexpression to increase KAT expression was abolished when silencing these PPARs (especially PPARα), a conclusion of a concerted action of the PPAR/PGC-1α system on KAT expression has been drawn [184]. These results have not been supported, however, at the metabolite level in a recent study finding no overall difference in KYNA levels in the liver and various brain regions of PGC-1α-knockout mice [185], leaving this issue open for further investigations. More recently, KYNA has been reported to increase PGC-1α expression by GPR35, promoting anti-inflammatory gene expression, suggesting another possible link between the two molecular systems [186]. 

### 3.2. In Vivo and Human Implications for a Protective role of the Kynurenine Pathway in EAE/MS

IDO is present and can be readily activated in various cell types in the CNS, including astrocytes and microglia, upon IFNγ stimulus [187], and it has likewise been found upregulated histologically in microglia/macrophages and activated functionally (as reflected by an elevated l-kynurenine/Trp ratio [188], and increased levels of QUIN [189,190] and 3-HK [190] in the CNS of EAE animals. The activation of IDO has been demonstrated in various autoimmune disorders and is considered to be an endogenous self-protective response; however, the sometimes toxic levels reached by known neurotoxic metabolites in EAE raises theoretical concerns as regards to the conditions in the CNS [151]. In support of a positive neuromodulatory role of IDO activation in EAE, genetic deficiency [191] or pharmacological blockage (evoked by 1-methyl-Trp) [187,188,192] has led to increased Th1 and Th17 responses, decreased Treg responses, and EAE exacerbation overall [191]. In line with these, the protective role of IDO activation in EAE has been demonstrated, as the administration of IFNγ-treated DCs provided symptomatic amelioration and decreased histopathological and MRI alterations of EAE in rats [193]. Similarly, estrogen administration induced IDO expression in DCs and led to concomitant T cell apoptosis, a mechanism that is proposed to explain estrogen-mediated EAE suppression and to at least in part underlie the decreased rate of relapses during pregnancy [194]. Demonstrating a potential crucial role of downstream kynurenine metabolites in IDO-mediated EAE suppression, the administration of 3-HAA resulted in an ameliorated EAE disease course with diminished Th1 and Th17 responses and an elevated Treg response, in part by an indirect action of DCs [191]. Similarly, cinnabarinic acid, a less well-studied endogenous kynurenine metabolite, was capable of protecting against EAE by enhancing Tregs at the expense of Th17 [195]. Treatment with *N*-(3,4,-dimethoxycinnamoyl) anthranilic acid (3,4-DAA), an orally active derivative 3-HAA analogue (also known as tranilast), likewise demonstrated a suppressive effect in EAE, with fewer and milder relapses observed in the treated animals [196]. The therapeutic potential of KYNA analogues with established neuroprotective potential [197] in EAE has not yet been demonstrated.

At the human level, evidence regarding IDO activation in MS has been seemingly controversial. Indeed, low Trp levels have first been documented in the plasma and CSF in MS patients under relapse, suggestive of IDO activation [198]. Others found increased l-kynurenine and *N*-formyl-kynurenine levels in the serum of MS patients, which may as well reflect IDO activation [199]. Subsequent early studies reported controversial results about CSF and serum Trp levels in MS [200,201]; however, a negative correlation was revealed between CSF levels of Trp and neopterin, a macrophage activity marker, during acute relapse, possibly representing IDO activation in CNS-infiltrating macrophages [200]. These results were followed by the failure of another group to detect a significant baseline difference in the plasma l-kynurenine/Trp ratio between relapsing-remitting MS and control samples; however, an increased l-kynurenine/Trp ratio was detected after treatment with INF-β, implicating IDO activation as a potential mode of action of INF-β products widely used in the first-line treatment of MS and clinically isolated syndrome (CIS) [202]. Similarly, before-treatment, IDO expression in PBMCs was comparable in acute MS relapse to that seen in healthy controls; however, significant IDO downregulation was observed due to glucocorticoid treatment of the relapse, and IDO expression in stable MS patients in remission was found to be decreased compared to healthy controls (these alterations were more or less well reflected also by the l-kynurenine/Trp ratio values measured from the serum) [203]. These results are almost identical with those reporting increased and decreased global AHR activity (reflecting decreased AHR agonist levels) in the serum during relapse and remission, respectively, implicating the role of the endogenous AHR agonist l-kynurenine, in particular [204]. A most recent publication reported an elevated l-kynurenine/Trp ratio in relapsing-remitting MS compared to healthy controls; however, the clinical phase of the patients during lumbar puncture (i.e., relapse or remission) is not clearly indicated [205]. These results together suggest that baseline IDO activity might be downregulated in stable MS, probably contributing to disease pathogenesis, whereas it is relatively upregulated during acute inflammatory relapse most probably reflecting an endogenous counter-regulatory reaction, which then responds to acute anti-inflammatory therapy (Figure 2). In line with all these, increased CSF QUIN levels and QUIN/l-kynurenine ratios exclusively under relapse have recently been reported [206]. Of notable similarity, plasma [156] and CSF [207] KYNA levels and KAT activity in the red blood cells [156] of relapsing-remitting MS patients have also been found to be elevated only during acute relapses, whereas during remission, CSF KYNA levels were low [208]. These together demonstrate increased downstream kynurenine metabolism in addition to IDO activity during an acute inflammatory exacerbation in MS (Figure 2). In addition, CSF KYNA levels tended to be low in secondary progressive MS patients [206], whereas serum KYNA levels have recently been found to be significantly decreased in both primary and secondary progressive MS patients compared to healthy controls, but not in patients with relapsing-remitting MS [205]. These findings were paralleled by significantly increased serum 3-HK and QUIN levels in all groups of MS in this study, yielding significantly increased serum QUIN/KYNA ratios in primary and secondary progressive MS groups [205]. The authors argue that this imbalance between neurotoxic and neuroprotective metabolites of the kynurenine pathway favoring the neurotoxic ones might contribute to neurodegeneration in progressive MS subtypes in part via NMDA receptor-mediated excitotoxicity [205].

### 3.3. Therapeutic and Diagnostic Perspectives of the Kynurenine Pathway in MS

Roquinimex, laquinimod, leflunomide, and its active metabolite, teriflunomide, are orally active immunomodulators with apparent structural and functional similarity to kynurenines, including anthranilic acid, arresting activated T cells and shifting the cytokine response toward Th2 [151]. These drugs were all highly effective in EAE [209,210,211,212].

Roquinimex (a.k.a. linomide) is a quinoline derivative that demonstrated high efficacy in EAE models in association with reduced lymphocyte proliferative responses [212]. After the promising preclinical era of linomide, the molecule was found to be associated with severe cardiotoxicity during its phase III clinical trials in humans, resulting in the cessation of clinical investigations [213]. 

Laquinimod, a.k.a ABR-215062 or Nerventra, is an orally administered quinoline-3-carboxamide, a derivative of linomide. Its immunomodulatory effect has been attributed to multiple mechanisms of action, including the activation of anti-inflammatory genes and the downregulation of proinflammatory genes, thereby reducing the number of proinflammatory immune cells and increasing the number of Tregs [214]. In relapsing-remitting MS, laquinimod was demonstrated to be capable of slowing disease progression and has been associated with both immunomodulatory (i.e., decreasing the relapse rate and the cumulative number of gadolinium enhancing CNS lesions) and neuroprotective features (i.e., reducing the progression of brain atrophy). Interestingly, laquinimod has demonstrated AHR agonistic activity [204], similarly to l-kynurenine and KYNA, and has a largely diminished effect on EAE in AHR knockout mice [209,210]. Despite the initial successes, the clinical investigation of laquinimod in relapsing-remitting MS has recently been stopped because of a number of failures, including cardiotoxicity in high doses and insufficient disease-modifying effects in low doses (CONCERTO trial, phase III, NCT01707992) [215].

Leflunomide, an isoxazole derivative, and its active metabolite, teriflunomide (a.k.a. A771726), possess in part similar effects and side effects. Teriflunomide, produced via the opening of the isoxazole ring, is considered to be safer and more effective than its precursor. Both leflunomide and teriflunomide were effective in EAE, and their effect is proposed to be predominantly attributable to the suppression of pyrimidine synthesis via the inhibition dihydroorotate dehydrogenase [211]. Though leflunomide is also known as an agonist of the AHR, interestingly, teriflunomide is proposed to have no such effect [216]. Recommended only in monotherapy, after succeeding in phase II and III clinical trials, teriflunomide has recently been approved and introduced as a first-line treatment for RRMS in the USA and the European Union in 2012 and 2013, respectively [217].

These data together support the potential of kynurenine derivatives (with high structural and functional similarity to the above molecules) in the treatment of MS and should encourage further research with the aim to develop analogues with more favorable clinical profiles. The above detailed alterations in IDO expression, IDO activity, various downstream l-kynurenine metabolite levels, as well as the global AHR activity in the acute phases of MS indicate their potential utility as biomarkers of underlying disease severity and surrogate markers of therapeutic response in future clinical trials.

## 4. Concluding Remarks

Endogenous immunomodulatory pathways and associated molecules have been linked to the development of MS and/or to activated compensatory mechanisms, and a number of different molecules have been implicated as potential biomarkers of MS or novel targets of its therapy. Emerging research in the last few years regarding PPARs, the experimentally highly potent endogenous immunomodulators, has delineated the probably predominant role of PPARγ among the isoforms both as a potential therapeutic target and as a biomarker in MS. While preliminary human data are reassuring, there is evidently a need for further examinations to well define the potential clinical benefits of PPARγ agonists in treatment and the potential role of PPARγ as a biomarker in MS. With the aim of exploiting the therapeutic benefits of Trp metabolites and their analogues, intensive research has been conducted, and a number of derivatives with high structural and functional resemblance to kynurenines have already approached the clinical level, leading to the introduction of the orally administered drug, teriflunomide, into the market of MS therapeutics, while cancelling others due to toxicity and/or inefficacy. The potential clinical role of IDO-related metabolic alterations as biomarkers in MS have yet to be established in future clinical studies. Endogenous immunomodulatory pathways merit extensive research in the context of MS, and further studies are encouraged to provide even more comprehensive knowledge of their complexity and to shed light on their possible clinical benefits.

## Figures and Tables

**Table 1:** Selected agonists of the different peroxisome proliferator-activated receptor (PPAR) isoforms and their implications in immunomodulation.

Isoform	Type	Molecule	Role	References
PPARα	synthetic	fenofibrate	Suppresses T cell proliferation and IL-1β, TNFα, and IL-6 production, and increases IL-4 production.	[27,28,29,30,31]
		gemfibrozil	Inhibits mononuclear cell infiltration and Th1 differentiation.	[27,28,30,31,32]
		WY14463	Inhibits IFNγ, IL-6, and TNFα production in T cells.	[27,28,30,31,33]
PPARγ	natural	15dPGJ2	Inhibits T cell proliferation, IL-1β, IL-4, IL-6, IL-10, IL-12, IFNγ, MCP1, NO, TNFα, and TLR4/TLR9 production, and Th1 differentiation.	[10,25,27,34,35,36]
	synthetic	GW7845	Reduces cytokine and chemokine secretion, and leukocyte infiltration.	[27,37]
		rosiglitazone	Reduces T cell infiltration into the brain.	[26,27,38]
		troglitazone	Suppresses IL1-β and TNFα.	[27,39]
		pioglitazone	Reduces INFγ and T cell response.	[27,35,40]
		ciglitazone	Inhibits IL-12 production of macrophage/microglial cells.	[27,29,41]
PPARβ/δ	synthetic	GW501516	Inhibits EAE by modulating the development of Th1 and Th17 responses and decreases the production of IFNγ and IL-17 in the CNS.	[11]
		GW610742	Reduces inflammation in the CNS.	[27,42,43,44]
		L-165041	Inhibits EAE by modulating the development of Th1 and Th17 responses and decreases the production of IFNγ and IL-17 in the CNS.	[11]

EAE: experimental autoimmune encephalomyelitis, IFNγ: interferon γ, IL: interleukin, MCP1: monocyte chemoattractant protein 1, NO: nitric oxide, PPAR: peroxisome proliferator-activated receptor, Th: T helper lymphocyte, TLR: Toll-like receptor, TNFα: tumor necrosis factor α.
